# 2-Acylamido Analogues of *N*-Acetylglucosamine Prime Formation of Chitin Oligosaccharides by Yeast Chitin Synthase 2*

**DOI:** 10.1074/jbc.M114.550749

**Published:** 2014-03-11

**Authors:** Jacob Gyore, Archana R. Parameswar, Carleigh F. F. Hebbard, Younghoon Oh, Erfei Bi, Alexei V. Demchenko, Neil P. Price, Peter Orlean

**Affiliations:** From the ‡ Department of Microbiology, University of Illinois at Urbana-Champaign, Urbana, Illinois 61801,; the § Departments of Chemistry and Biochemistry, University of Missouri, St. Louis, Missouri 63121,; the ¶ Department of Cell and Developmental Biology, University of Pennsylvania Perelman School of Medicine, Philadelphia, Pennsylvania 19104, and; ‖ Renewable Product Technology, National Center for Agricultural Utilization Research, Agricultural Research Service, United States Department of Agriculture, Peoria, Illinois 61604

**Keywords:** Carbohydrate Biosynthesis, Chitin, Glycosyltransferases, Polysaccharide, Yeast Physiology

## Abstract

Chitin, a homopolymer of β1,4-linked *N*-acetylglucosamine (GlcNAc) residues, is a key component of the cell walls of fungi and the exoskeletons of arthropods. Chitin synthases transfer GlcNAc from UDP-GlcNAc to preexisting chitin chains in reactions that are typically stimulated by free GlcNAc. The effect of GlcNAc was probed by using a yeast strain expressing a single chitin synthase, Chs2, by examining formation of chitin oligosaccharides (COs) and insoluble chitin, and by replacing GlcNAc with 2-acylamido analogues of GlcNAc. Synthesis of COs was strongly dependent on inclusion of GlcNAc in chitin synthase incubations, and *N*,*N*′-diacetylchitobiose (GlcNAc_2_) was the major reaction product. Formation of both COs and insoluble chitin was also stimulated by GlcNAc_2_ and by *N*-propanoyl-, *N*-butanoyl-, and *N*-glycolylglucosamine. MALDI analyses of the COs made in the presence of 2-acylamido analogues of GlcNAc showed they that contained a single GlcNAc analogue and one or more additional GlcNAc residues. These results indicate that Chs2 can use certain 2-acylamido analogues of GlcNAc, and likely free GlcNAc and GlcNAc_2_ as well, as GlcNAc acceptors in a UDP-GlcNAc-dependent glycosyltransfer reaction. Further, formation of modified disaccharides indicates that CSs can transfer single GlcNAc residues.

## Teaser

**Background:** Chitin synthases are stimulated by *N*-acetylglucosamine (GlcNAc).

**Results:** GlcNAc and 2-acylamido analogues of GlcNAc stimulate formation of chitin oligosaccharides by yeast chitin synthase, and GlcNAc is transferred to the 2-acylamido analogues.

**Conclusion:** Chitin synthases use GlcNAc analogues as primers and transfer one GlcNAc at a time.

**Significance:** Results are new insights into polysaccharide synthase mechanism and suggest ways of synthesizing novel modified polysaccharides.

## Introduction

Chitin, a homopolymer of β1,4-linked GlcNAc residues, is a key component of fungal cell walls and the arthropod exoskeleton. The polymer is synthesized by processive inverting enzymes of glycosyltransferase family 2, whose members include cellulose synthases and class I hyaluronic acid synthases (1–5). The chitin synthase (CS)^2^ reaction is typically described as transfer of GlcNAc from UDP-GlcNAc to preexisting chitin chains. Fungi usually have multiple CSs, which can be active in different cellular locations and at different times during cell growth and division (6–8).

CSs are commonly assayed by incubating membrane fractions or partially purified enzyme preparations with UDP-GlcNAc and then collecting insoluble product for quantification (9–12). *In vitro* activity of CSs in membranes is often stimulated up to a few -fold by the inclusion of free GlcNAc in incubations (10, 12–16), and GlcNAc has been suggested to serve as a co-substrate (14) or an allosteric activator (16) in the CS reaction. Consistent with GlcNAc serving as a co-substrate or primer, the bacterial CS homologue NodC can transfer GlcNAc from UDP-GlcNAc to the nonreducing end of free GlcNAc, as well as transfer GlcNAc to the nonreducing end of *p*-nitrophenyl β-GlcNAc (17). A primer function for free GlcNAc, however, has not been demonstrated for a eukaryotic CS. Preexisting chitin chains presumably serve as acceptors for further GlcNAc addition, but short chitin oligosaccharides (COs) do not, because NodC did not use short COs as GlcNAc acceptor from UDP-GlcNAc (17), nor did a purified yeast CS (Chs1) elongate chitotetraose into chitin in the presence of UDP-GlcNAc (9). However, it has been proposed that longer COs can serve as primers (18).

In this study, we explore the role of GlcNAc in detail by using an *Saccharomyces cerevisiae* strain that expresses a single CS, by examining low molecular weight reaction products and by studying the effects of the GlcNAc analogues *N*-propanoyl- (GlcNPr), *N*-butanoyl- (GlcNBu), and *N*-glycolyl- (GlcNGc) substituted glucosamine. We show that formation of COs is strongly dependent on the inclusion of free GlcNAc or certain 2-acylamido analogues of GlcNAc in assays and that Chs2 can transfer a single GlcNAc from UDP-GlcNAc to 2-acylamido analogues of GlcNAc and extend the resulting disaccharide with further GlcNAc residues.

## EXPERIMENTAL PROCEDURES

### 

#### 

##### Yeast Strains and Culture Media

*S. cerevisiae* strains YO1111 (*chs1*Δ *chs3*Δ), YO1528 (*chs1*Δ *chs3*Δ pRS314), and YO1535 (*chs1*Δ *chs3*Δ pYO201 (*CHS2* overexpresser)) were described previously (19). The *CTS1* gene in YO1535 was deleted and replaced with the yeast *LEU2* gene (20). The sequence of the forward oligonucleotide primer used to amplify a DNA fragment consisting of *LEU2* and nucleotides immediately upstream and downstream of the *CTS1* coding sequence was 5′-ACATTGAAATTCTAATTTAAATATAAAATAATTAATAATAGAATGCGTTTCGGTGATGAC-3′, and the sequence of the reverse primer was 5′-ACATTGAAATTCTAATTTAAATATAAAATAATTAATAATAGAATGCGTTTCGGTGATGAC-3′. Elimination of Cts1 activity was verified by testing culture supernatants of candidate *cts1::LEU2* mutants for release of 4-methylumbelliferone from 4-methylumbelliferyl-β-d-*N*,*N*′,*N*″-triacetylchitotrioside (Sigma) (21). For induction of *CHS2* expression, strains were pregrown for 24 h at 30 °C in synthetic complete medium lacking tryptophan containing 2% (w/v) glucose. Cells were then collected by centrifugation and resuspended in 250 ml of synthetic complete medium minus tryptophan containing 2% (w/v) galactose and 1% (w/v) raffinose. Induction of *CHS2* expression was performed for 18–21 h at 25 °C.

##### Membrane Preparation

Mixed membrane fractions were prepared as described (19) with the exception that glycerol was omitted from the final buffer (30 mm Tris-HCl, pH 7.5) in which membranes were homogenized. Membranes were frozen at −80 °C and thawed just before use.

##### Assays for Chitin and Chitin-Oligosaccharide Synthesis

Incubation mixtures for assay of formation of 10% trichloroacetic acid-insoluble, [^14^C]GlcNAc-labeled polymer contained, in a final volume of 50 μl of 2 mm UDP-GlcNAc, 50 nCi of UDP-[^14^C]GlcNAc (specific activity 300 mCi/mmol; American Radiolabeled Chemicals, St. Louis, MO), 2.5 mm cobalt acetate, and, when included, 32 mm GlcNAc, *N*,*N*′-diacetylchitobiose, or *N*,*N*′,*N*″*-*triacetylchitotriose (from Sigma) (19). Reactions were started by addition of 20 μl of membranes, and mixtures were incubated at 30 °C, typically for 30 min. Incubation mixtures for formation of COs contained 125 or 250 nCi of UDP-[^14^C]GlcNAc, corresponding to final UDP-GlcNAc concentrations of 0.046 mm or 0.092 mm, respectively. In some assays of CO synthesis, unlabeled UDP-GlcNAc (from Sigma) was added to give higher final UDP-GlcNAc concentrations. Reaction mixtures were then fractionated according to an adaptation of the procedure of Bligh and Dyer (22). To stop reactions, 375 μl of chloroform/methanol 1:2 (v/v) was added to incubation tubes (22). After standing for 30 min at room temperature, 125 μl of chloroform and 50 μl of water were added to the tubes, which were then mixed by vortexing and centrifuged in a Microfuge for 15 min. The upper aqueous phase was transferred to a minicolumn containing approximately 0.5 ml of packed Dowex 1-X8 resin (200–400 mesh) and the column run-through collected. The column was washed twice with 250 μl of water and once with 250 μl of 50% aqueous ethanol, and the combined run-throughs were dried under a stream of air. In some experiments, the insoluble material remaining after chloroform/methanol/water extraction was precipitated in 10% TCA after removal of the organic phase. COs were separated by thin layer chromatography (TLC) on Silica Gel 60 plates that had been prerun in chloroform/methanol/water (65:25:4 v/v/v). Chromatograms were developed twice in butan-1-ol/ethanol/water (5:3:2 v/v/v), and radiolabeled material was detected by phosphorimaging. Nonradioactive standards of GlcNAc, *N*,*N*′-diacetylchitobiose, and *N*,*N*′,*N*″-triacetylchitotriose were detected by spraying with aniline-diphenylamine-phosphoric acid reagent (23).

For bulk preparation of unlabeled COs, incubation mixtures (50-μl final volume) contained 1.4 mm unlabeled UDP-GlcNAc and 0.25 mm cobalt acetate. The Dowex 1-X8 run-throughs from seven parallel incubations were pooled, evaporated to dryness, and submitted to charcoal-celite chromatography as follows. Minicolumns were prepared by loading a 5-ml disposable pipette tip with a slurry of equal amounts of activated charcoal and celite 545 in 5% aqueous ethanol to give a column bed of 5 cm. CO samples were dissolved in 1 ml of water and loaded onto the column, which was then washed with 10 ml of 5% aqueous ethanol, and the eluate collected. The column was then eluted with 25 ml of 30% aqueous ethanol, and five 5-ml fractions were collected. Analysis of the fractions by MALDI established that free GlcNAc and salt emerged predominantly in the 5% ethanol wash and in 30% ethanol fraction 1, whereas the COs were eluted predominantly in 30% ethanol, in fractions 2 and 3 (24).

##### Matrix-assisted Laser Desorption/Ionization–Time-of-flight (MALDI-TOF) Mass Spectrometry

MALDI-TOF mass spectra were recorded on a Bruker Daltonics Microflex LRF instrument (Bruker-Daltonics, Billerica, MA) operating in reflectron mode. The system utilizes a pulsed nitrogen laser, emitting at 337 nm. Typically, 1000–2000 shots were acquired at 60-Hz frequency and 78% laser power, with the laser attenuator offset at 16% for 30% range. The matrix was saturated 2,5-dihydrobenzoic acid in acetonitrile and was premixed with the samples (0.5 mg·ml^−1^) prior to spotting onto a standard 96-position stainless steel target. Ion source 1 was set to 19.0 kV, and source 2 to 15.9 kV (83.7% of IS 1), with lens and reflector voltages of 9.79 and 19.99 kV, respectively. During the acquisition matrix ion suppression was used up to 200 Da. External calibration used Bruker Peptide Calibration Standard II mono with insulin. The MS data were processed off-line using the Flex Analysis 3.0 software package (Bruker Daltonics).

##### Preparation of 2-Acylamido Analogues of GlcNAc

Routine procedures and sources of reagents were as follows. Column chromatography was performed on Silica Gel 60 (70–230 mesh), reactions were monitored by TLC on Kieselgel 60 F254, and compounds were detected by examination under UV light and by charring with 10% sulfuric acid in methanol. Solvents were removed under reduced pressure at <40 °C. d-Glucosamine·HCl, propanoic anhydride, butyric anhydride, acetoxyacetyl chloride, anhydrous pyridine, anhydrous methanol, and inorganic compounds were purchased from Sigma-Aldrich and used as is. ^1^H and ^13^C NMR spectra were recorded in D_2_O at 300 MHz or 75 MHz (Bruker Avance), respectively.

GlcNPr and GlcNBu were prepared from d-glucosamine·HCl as described previously, and the analytical data for these compounds were practically the same as reported previously (25). For the preparation of GlcNGc, d-glucosamine·HCl (5.0 g, 23.2 mmol) was dissolved in cold water (25 ml), and NaHCO_3_ (5.8 g, 69.5 mmol) was added. The mixture was stirred vigorously in an ice bath, and acetoxyacetyl chloride (3.0 ml, 27.8 mmol) was added dropwise. The resulting mixture was stirred for additional 2 h in the ice bath, then neutralized by dropwise addition of 1 m HCl. The precipitate was filtered off, washed with ice-cold water (10 ml), and dried. The crude product (∼10 g) was dissolved in pyridine (50 ml) and acetic anhydride (25 ml) was added. The reaction mixture was stirred for 16 h at room temperature, then quenched by addition of methanol (∼20 ml), and the volatiles were removed under reduced pressure. The residue was dissolved in CH_2_Cl_2_ (300 ml), and the organic phase was washed successively with water (200 ml), saturated aqueous NaHCO_3_ (200 ml), water (200 ml), 1 m HCl (2 × 200 ml), and finally, with water (2 × 200 ml). The organic phase was separated, dried with MgSO_4_, and concentrated *in vacuo*. The residue was purified by column chromatography on silica gel (methanol-dichloromethane gradient elution). The pure acetylated product (α-anomer, 2.3 g) was dissolved in methanol (5 ml), and 1 m sodium methoxide in methanol (2.5 ml) was added, giving a pH of 9, and the reaction mixture was stirred for 48 h at room temperature. The resulting mixture was neutralized with Dowex (H^+^), the resin was filtered off and rinsed with methanol. The combined filtrate was concentrated *in vacuo* and dried. The residue was purified by column chromatography on silica gel (methanol-dichloromethane gradient elution) to afford GlcNGc (0.8 g) in 15% yield overall. Selected analytical data for GlcNGc are: ^13^C NMR (α-anomer): δ, 53.6, 60.5, 60.8, 69.9, 70.7, 71.6, 90.8, 175.1 ppm; ^13^C NMR (β-anomer): δ, 56.3, 60.7, 61.0, 69.8, 73.6, 75.9, 94.6, 175.6 ppm. The remaining analytical data were essentially the same as reported previously (26, 27).

## RESULTS

To explore the effect of free GlcNAc on the activity of a single CS, we used an *S. cerevisiae chs1*Δ *chs3*Δ strain, which lacks two of the three CS activities of yeast but is viable because it retains its chromosomal copy of the gene for the remaining CS, Chs2. The activity of chromosomally encoded Chs2 in membranes from the *chs1*Δ *chs3*Δ strain grown in minimal medium is very low, and *in vitro* Chs2 activity only becomes detectable when *CHS2* is overexpressed from a high copy, galactose-inducible plasmid (15, 19). Although Chs2 activity can be elevated by pretreating membranes with trypsin (10), membranes from the present *CHS2*-overexpressing strain have high Chs2 activity without prior trypsin treatment (19), and the experiments here were done without trypsin treatment of membranes. To determine the nature of Chs2 reaction products at higher resolution, we focused on COs, which are made by *S. cerevisiae* CSs at low UDP-GlcNAc concentrations (9, 12, 28).

### 

#### 

##### GlcNAc Strongly Stimulates Formation of GlcNAc_2_ and COs

**FIGURE 1.**
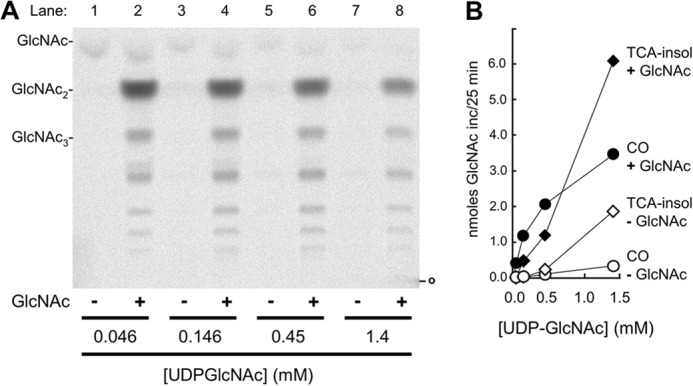
**Stimulation of CO and insoluble chitin synthesis by GlcNAc.** *A*, CO synthesis. Chs2-overexpressing membranes were incubated with 125 nCi of UDP-[^14^C]GlcNAc and increasing amounts of unlabeled UDP-GlcNAc in the presence or absence of 32 mm GlcNAc. Aqueous-soluble reaction products were isolated, passed through Dowex 1-X8 resin, and separated by TLC on silica gel plates that were developed twice in butan-1-ol/ethanol/water (5:3:2 v/v/v), after which radiolabeled material was detected by phosphorimaging, as detailed under “Experimental Procedures.” Positions of nonradioactive standards are indicated. *B*, quantification of COs and chitin. Total amounts of COs in the material that was applied to the chromatogram in *A* and total amounts of 10% TCA-insoluble (*TCA-insol*) chitin remaining after chloroform/methanol/water extraction of the incubation mixtures from *A* were determined and plotted against UDP-GlcNAc concentration.

Chs2-overexpressing membranes from *chs1*Δ *chs3*Δ cells were incubated with fixed amounts of UDP-[^14^C]GlcNAc and increasing amounts of unlabeled UDP-GlcNAc, and reaction mixtures were then fractionated into aqueous-soluble, organic-soluble, and chloroform/methanol/water-insoluble material according to an adaptation of the procedure of Bligh and Dyer (22). The aqueous-soluble material obtained after chloroform/methanol/water extraction and phase partitioning was passed through Dowex 1-X8, material in the run-through separated by TLC, and radiolabeled material on the TLC plate detected by phosphorimaging. This experiment yielded the following findings. First, CS incubations done in the presence of 32 mm GlcNAc generated material with chromatographic mobilities similar to those of the GlcNAc_2_ and GlcNAc_3_ standards, as well as more nonpolar material (Fig. 1*A*, *lanes 2*, *4*, *6*, and *8*), whereas only traces of radiolabeled bands were generated in incubations carried out in the absence of free GlcNAc (Fig. 1*A*, *lanes 1*, *3*, *5*, and *7*). The profiles of radiolabeled bands formed in the presence of GlcNAc resembled those reported previously for COs (28), and radiolabeled material in the Dowex run-through will henceforth be referred to as COs. Second, quantification of the COs and the insoluble material remaining after Bligh-Dyer extraction revealed that larger amounts of COs were made with increasing UDP-GlcNAc concentration in the presence of GlcNAc and that increasing amounts of insoluble material were made in both the presence and absence of GlcNAc (Fig. 1*B*). Indeed, at 1.4 mm UDP-GlcNAc, more than half of the reaction products formed in the presence of free GlcNAc were COs. Third, GlcNAc strongly stimulated formation of radiolabeled material that co-migrated with GlcNAc_2_, suggesting that the disaccharide is the major CS product. However, this apparent stimulation of GlcNAc_2_ formation may in part reflect a less than quantitative recovery of larger COs with the present procedure.

Formation of COs was dependent on overexpression of *CHS2* because the CO fraction obtained after incubation of membranes from the control *chs1*Δ *chs3*Δ *CHS2* strain, which makes negligible amounts of insoluble chitin (15, 19), contained no detectable radiolabeled COs, irrespective of whether free GlcNAc was included in the incubations or not. Further, the COs are unlikely to be generated postsynthetically by the action of yeast chitinase (a possibility raised by Kang *et al.* (9)) because deletion of the yeast endochitinase gene *CTS1* in our overexpression host was without effect on CO formation.

**FIGURE 2.**
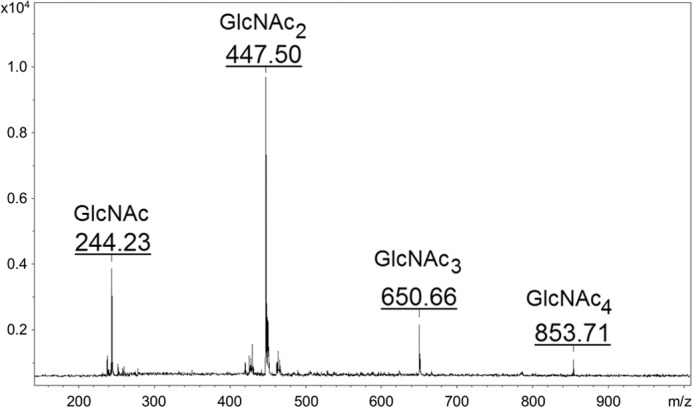
**Mass spectrometric analysis of COs made in the presence of GlcNAc.** Membranes from YO1535 *cts1*Δ cells overexpressing *CHS2* were incubated with 1.4 mm unlabeled UDP-GlcNAc, and pooled CO fractions generated in seven replicate incubations were chromatographed on activated charcoal-celite, concentrated, and submitted to MALDI-TOF mass spectrometry. Indicated masses are those of sodium adduct [M + Na]^+^ ions.

The fact that incubations performed with 1.4 mm unlabeled UDP-GlcNAc yielded larger amounts of COs gave us an opportunity to isolate amounts of unlabeled COs sufficient for analysis by MALDI. The CO fraction from incubations of membranes from the *CHS2*-overexpressing strain carried out with 32 mm GlcNAc contained peaks whose masses correspond to those of the sodium adduct [M + Na]^+^ ions of GlcNAc_2_, GlcNAc_3_, and GlcNAc_4_ (Fig. 2), whereas the corresponding fraction from incubations with control strain YO1528 harboring only the chromosomal copy of *CHS2* did not contain detectable peaks corresponding to these masses. This finding confirmed that the CO fraction from incubations containing 32 mm GlcNAc contained GlcNAc oligosaccharides whose formation is dependent on overexpression of *CHS2*. The CO fraction from incubations carried out without free GlcNAc contained insufficient material for analysis.

**FIGURE 3.**
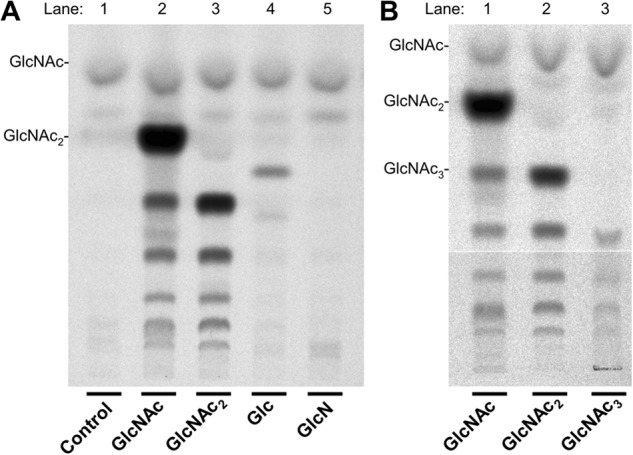
**Effects of GlcNAc_2_, GlcNAc_3_, glucose, and GlcN on CO formation.** *A*, Chs2-overexpressing membranes that had been resuspended in buffer lacking glycerol were assayed for CO formation in the presence of 32 mm GlcNAc, GlcNAc_2_, Glc, and GlcN as described for Fig. 1*A. B*, stimulation of CO formation by GlcNAc_1–3_. Incubations were performed in the presence of 32 mm GlcNAc, GlcNAc_2_, and GlcNAc_3_ as described for *A*.

We also tested GlcNAc_2_, GlcNAc_3_, Glc, GlcN, GalNAc, and ManNAc for their effect on CO synthesis. GlcNAc_2_ stimulated formation of material with the same chromatographic mobility as GlcNAc_3_, as well as more polar COs with the same mobility as those made in the presence of GlcNAc (Fig. 3*A*, *lanes 2* and *3*; Fig. 3*B*, *lanes 1* and *2*). Inclusion of GlcNAc_3_ in incubations led to formation of material with chromatographic mobilities similar to those of GlcNAc_4_ and larger COs (Fig. 3*B*, *lane 3*) although recovery of COs was poor, possibly because of low solubility of GlcNAc_3_ in the incubation mixture. In the presence of Glc, a small amount of material with a mobility between those of GlcNAc_2_ and GlcNAc_3_ was formed (Fig. 3*A*, *lane 4*), whereas GlcN (Fig. 3*A*, *lane 5*), GalNAc, and ManNAc were without effect. Stimulation of insoluble chitin synthesis by Glc but not by GlcN has been noted (13, 16). One possible explanation for the new material formed in the presence of Glc is that it is a disaccharide of GlcNAc and Glc. We also noted that when glycerol was present in high concentrations, it was a potential acceptor substrate for Chs2. The finding that GlcNAc_2_ stimulates formation of ^14^C-labeled GlcNAc_3_ and larger COs, but not [^14^C]GlcNAc_2_ synthesis, raised the possibility that GlcNAc_2_, might prime, rather than activate CO formation.

##### Stimulation of CO and Chitin Synthesis by 2-Acylamido-GlcNAc Derivatives

**FIGURE 4.**
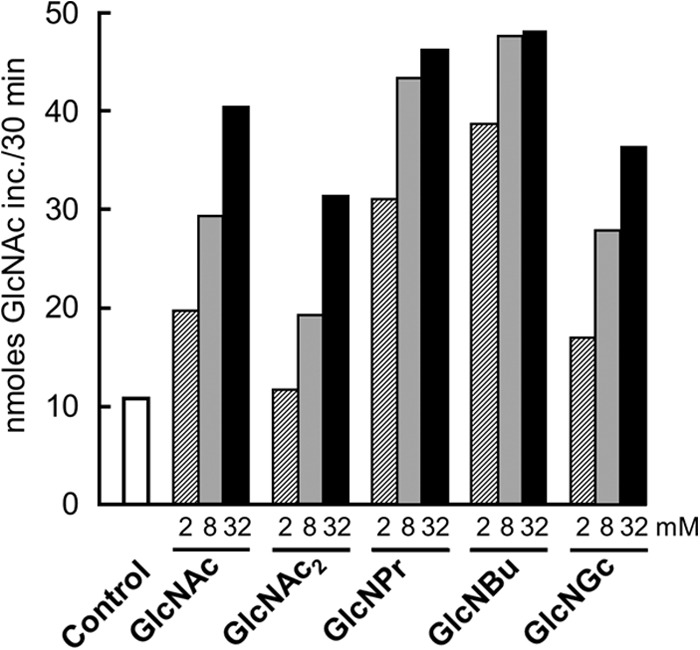
**Stimulation of chitin synthesis by 2-acylamido-GlcNAc analogues.** Chs2-overexpressing membranes were assayed for formation of 10% TCA-insoluble chitin in the presence of the indicated concentrations of GlcNAc_2_, GlcNPr, GlcNBu, and GlcNGc.

**FIGURE 5.**
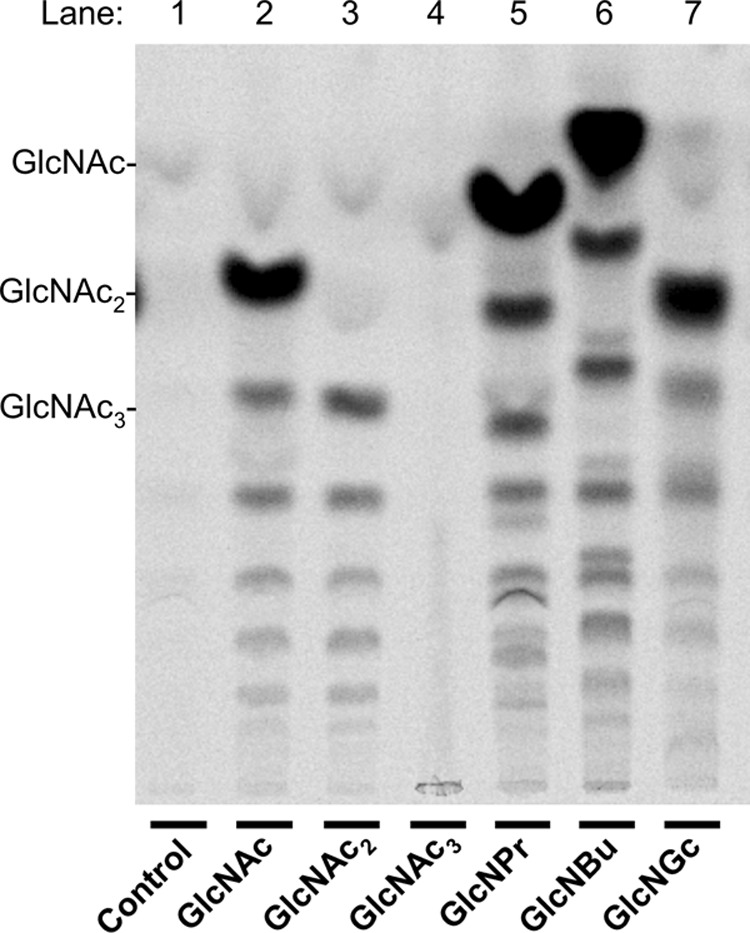
**Stimulation of CO formation synthesis by 2-acylamido-GlcNAc analogues.** Chs2-overexpressing membranes were assayed for CO formation in the presence of GlcNAc_2_, GlcNAc_3_, GlcNPr, GlcNBu, and GlcNGc, all at 32 mm. TLC was performed as for Fig. 1*A*. In this experiment, GlcNAc_3_ did not stimulate CO formation.

**FIGURE 6.**
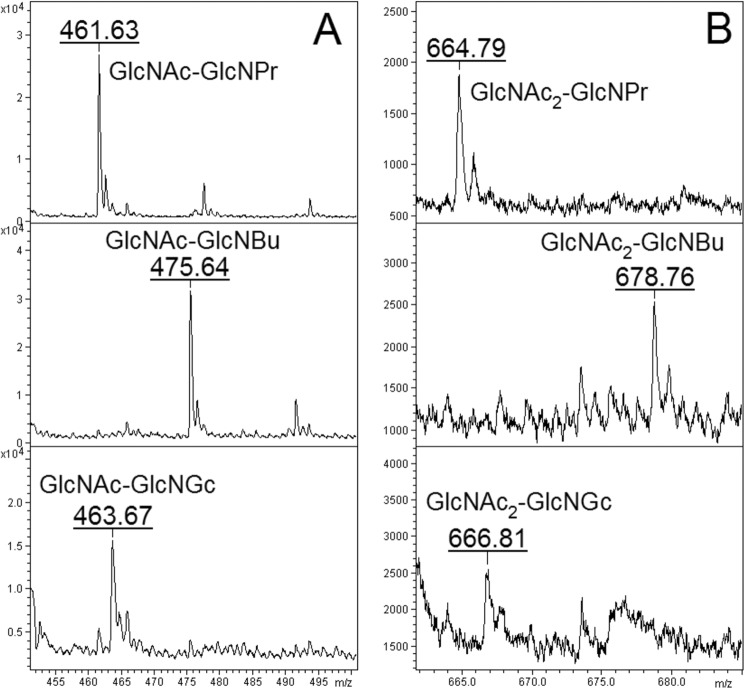
**Formation of COs in the presence of GlcNPr, GlcNBu, and GlcNGc.** Membranes from YO1535 *cts1*Δ cells overexpressing *CHS2* were incubated with 1.4 mm unlabeled UDP-GlcNAc and 32 mm GlcNPr, GlcNBu, or GlcNGc, and pooled CO fractions generated in seven replicate incubations were chromatographed on activated charcoal-celite, concentrated, and submitted to MALDI. *A*, material in *m*/*z* range of disaccharides. *B*, material in *m*/*z* range of trisaccharides.

**FIGURE 7.**
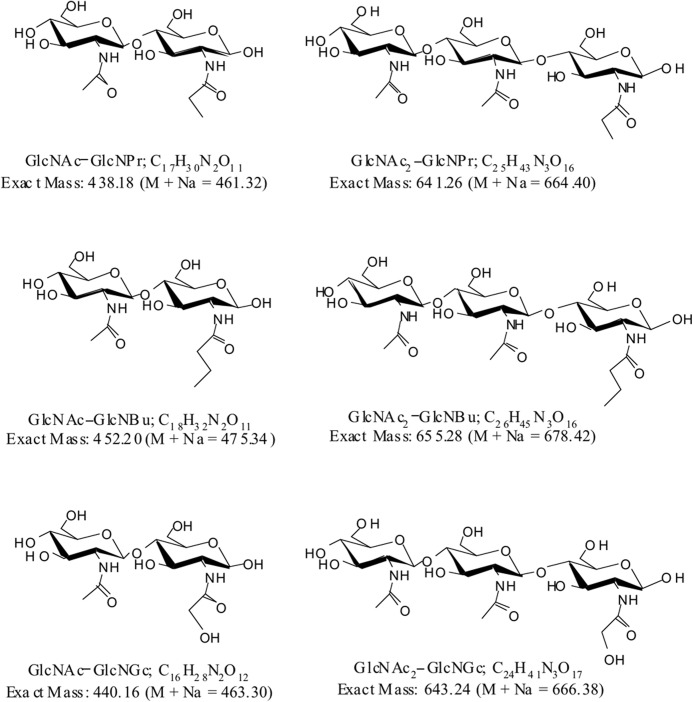
**Calculated masses and molecular formulae for the variously N***-***acylated CO disaccharide and trisaccharide sodium adduct [M + Na]^+^ ions observed by MALDI-TOF mass spectrometry.** The mass spectra are shown in Fig. 6. Structures are drawn assuming nonreducing end addition of GlcNAc.

Stimulation of GlcNAc_2_ and CO synthesis could be explained either by GlcNAc serving as an activator of the enzyme or as a priming substrate, but these possibilities would not be distinguished if the masses of the reaction products were determined. However, if a GlcNAc analogue could substitute for GlcNAc, then only if that analogue were a GlcNAc acceptor would the mass of the product di- and oligosaccharides change. The availability of *N*-propanoyl-, *N*-butanoyl-, and *N*-glycolylglucosamine (GlcNPr, GlcNBu, and GlcNGc) made this experiment possible. All three GlcNAc analogues, as well as GlcNAc_2_, stimulated chitin formation in the standard assay for 10% TCA-insoluble chitin formation with 2 mm UDP-GlcNAc (Fig. 4). Indeed, at the three concentrations tested, GlcNPr and GlcNBu gave stronger stimulation than GlcNAc itself did. Further, GlcNPr, GlcNBu, and GlcNGc all stimulated formation of ^14^C-labeled species that resembled the ladder of COs made in the presence of GlcNAc, but whose chromatographic mobilities were systematically shifted in a manner consistent with the possibility that the analogues had been incorporated into COs (Fig. 5). MALDI analysis of the Dowex run-throughs containing material made in the presence of GlcNPr, GlcNBu, and GlcNGc confirmed this, revealing the presence of material with masses expected for sodium adduct [M + Na]^+^ ions of disaccharides of each GlcNAc analogue and a single GlcNAc, and of material with masses expected for sodium adduct [M + Na]^+^ ions of trisaccharides containing each GlcNAc analogue and GlcNAc_2_ (Figs. 6 and 7). Because the GlcNAc analogues had been incorporated into modified COs, they must have served as acceptors for GlcNAc transfer by Chs2. The finding that modified disaccharides were made (and indeed were a major product) indicates that Chs2 transferred a single GlcNAc from UDP-GlcNAc.

## DISCUSSION

By focusing on the soluble products of the reaction carried out by yeast Chs2, we have obtained new insights into the synthetic capabilities of CSs. Our major findings are that (i) *in vitro* formation of COs by Chs2 is strongly dependent on free GlcNAc and the 2-acylamido GlcNAc analogues tested; (ii) Chs2 transfers GlcNAc from UDP-GlcNAc to the GlcNAc analogues GlcNPr, GlcNBu, and GlcNGc; and (iii) Chs2 can transfer single GlcNAc residues yielding a disaccharide as major product. Our results are the first direct evidence that a eukaryotic chitin synthase can use a low molecular weight primer. The formation of COs by yeast CSs has been documented (9, 12, 23), but in these studies, free GlcNAc was always included in the incubations, masking the extent to which the monomer stimulates CO formation. Because GlcNPr, GlcNBu, and GlcNGc all serve as GlcNAc acceptors, we propose that free GlcNAc, GlcNAc_2_, and GlcNAc_3_ do as well.

Our findings suggest that at least part of the stimulatory effect of free GlcNAc on chitin synthesis may be because of its acting as an acceptor for GlcNAc transfer, but we cannot rule out the possibility that GlcNAc has an additional role as allosteric activator of CO and insoluble chitin synthesis. However, because GlcNAc_2_, GlcNPr, GlcNBu, and GlcNGc did not stimulate formation of unmodified GlcNAc_2_ or GlcNAc_3_, a role as generic activator of CO synthesis would have to be restricted to GlcNAc itself.

Horsch *et al.* (16) used *Mucor rouxii* CS preparations and GlcNAc analogues to probe the structural requirements for activation and concluded that an aminoglucopyranose skeleton with an acylated amino group and a single-bonded oxo function at C-1 were necessary for the compound to act as an effector. These authors did not report whether the stimulatory GlcNAc analogues were incorporated into CS product. Our results with the 2-acylamido analogues of GlcNAc indicate that yeast Chs2 can use these analogues as acceptors and therefore that the enzyme tolerates bulkier substituents at C-2 of acceptor GlcNAc residues. Large groups at the C-6 position may also be tolerated because addition of 6-*O*-dansyl-GlcNAc to regenerating *Candida albicans* spheroplasts led to incorporation of this GlcNAc analogue into alkali-insoluble material (29), although it is not certain that a CS was directly involved.

It is not clear how GlcNAc-stimulated CO formation, the primer function of GlcNAc and its analogues, and GlcNAc-dependent stimulation of insoluble chitin synthesis are all related to the mechanism of chitin formation by Chs2 *in vitro*. We consider possible explanations in the context of processive and distributive mechanisms for polysaccharide polymerization.

The current model for the polymerization mechanism of glycosyltransferase family 2 polysaccharide synthases, which is based on the structure of *Rhodobacter* cellulose synthase BcsA, is for chain extension one sugar residue at a time with concomitant extrusion of the growing glycan chain through a channel created by the transmembrane domains of the enzyme (30). In the context of this processive model, the COs formed by Chs2 in the presence of GlcNAc may be generated as a result of premature chain termination (9), but alternatively, they may result from aberrant initiation *in vitro*. Thus, GlcNAc and its analogues may intrude into the catalytic site, compete with an enzyme-bound, nascent chitin chain, and prime CO formation, whereupon some COs dissociate from the enzyme, but others remain bound and are elongated, explaining the stimulatory effect of GlcNAc and its analogues on synthesis of both COs and insoluble chitin. This speculative explanation accommodates preliminary observations that the COs formed in pulse-chase experiments appeared stable^3^ (9) and leads to the prediction that average length of the *in vitro* Chs2 products formed in the presence of GlcNAc will be shorter than the product made in the absence of GlcNAc.

It is formally possible that Chs2 uses a distributive polymerization mechanism, in which the synthase disengages from its elongated product after every round of catalysis, then reassociates with a new acceptor and donor substrates for transfer of another monomer (31). In this case, free GlcNAc would also be expected to enhance CO formation.

The finding that GlcNAc stimulates CO and chitin synthesis *in vitro* is consistent with GlcNAc being the normal primer of *de novo* chitin synthesis, but we cannot exclude the possibility that *in vitro*, GlcNAc and its 2-acylamido analogues mimic an endogenous primer that is distinct from GlcNAc. If the latter is the case, this primer moiety should be present on the COs made in the absence of free GlcNAc, but because the amounts of COs made in these incubations are too small for analysis, it is not yet possible to determine whether these COs bear a terminal moiety different from GlcNAc. If free GlcNAc is indeed the *in vivo* primer, it would have to be generated by dephosphorylation of GlcNAc phosphates formed during UDP-GlcNAc synthesis or following hydrolysis of UDP-GlcNAc because the free sugar is not an intermediate in UDP-GlcNAc synthesis (32, 33).

Our findings were made with *S. cerevisiae* Chs2 and with membranes that had not been pretreated with protease, but we propose they apply to other CSs as well. However, CSs may differ in their relative abilities to use GlcNAc and its 2-acylamido analogues as acceptors, as well as in the extent to which these compounds stimulate CO formation *in vitro*. Partial proteolysis may also impact the response of CSs to GlcNAc and its 2-acylamido analogues, as well as the size range of CS products.

Our results have implications for the mechanism of other β-linked polysaccharide synthases of glycosyltransferase family 2. The finding that Chs2 can transfer a single GlcNAc from UDP-GlcNAc is direct support for the conclusion drawn from the structure-based model for the bacterial cellulose synthase BcsA that spatial restrictions in the substrate binding site would allow cellulose extension by one, rather than two glucoses at a time (30). Kamst *et al.* (17) also concluded that the bacterial chitin synthase homologue NodC sequentially transferred monosaccharides during CO synthesis. The finding that the 2-acetylamido position can tolerate modifications raises the possibility of introducing reactive groups at this position to tether acceptor residues. Further, CSs may prove able to use the UDP-derivatives of 2-acylamido GlcNAc analogues as substrates and generate chitin derivatives whose 2-acylamido side chains bear groups that confer novel properties.

## Footnotes

3 P. Orlean, unpublished observation.

## Footnotes

2 The abbreviations used are:

CS: chitin synthase
CO: chitin oligosaccharide
GlcNAc_2–4_: *N*,*N*′-diacetylchitobiose, *N*,*N*′,*N*″-triacetylchitotriose, and *N*,*N*′,*N*″,*N*‴-tetraacetylchitotetarose, respectively
GlcNBu: *N*-butanoylglucosamine
GlcNGc: *N*-glycolylglucosamine
GlcNPr: *N*-propanoylglucosamine.
